# Equilibrium and kinetic studies of copper biosorption by dead *Ceriporia lacerata* biomass isolated from the litter of an invasive plant in China

**DOI:** 10.1186/s40201-015-0191-1

**Published:** 2015-04-25

**Authors:** Xiaona Li, Airong Li, Mingzhong Long, Xingjun Tian

**Affiliations:** School of Life Science, Nanjing University, Nanjing, 210093 China; Institute of South China Karst, Guizhou Normal University, Guiyang, 550001 China; Co-Innovation Center for Sustainable Forestry in Southern China, Nanjing Forestry University, Nanjing, 210037 China; The State Key Laboratory Incubation Base for Karst Mountain Ecology Environment of Guizhou Province, Guizhou Normal University, Guiyang, 550001 China; School of Pharmaceutical Sciences, Zhengzhou University, Zhengzhou, 450001 China; Research Center for Karst Wetland Ecology, Guizhou Minzu University, Guiyang, 550025 China

**Keywords:** *Ceriporia lacerata*, Biosorption, Copper, Adsorption isotherm, Kinetics

## Abstract

**Background:**

*Ceriporia lacerata*, a strain of white-rot fungus isolated from the litter of an invasive plant (*Solidago canadensis*) in China, was little known about its properties and utilization. In this work, the copper(II) biosorption characteristics of formaldehyde inactivated *C. lacerata* biomass were examined as a function of initial pH, initial copper(II) concentration and contact time, and the adsorptive equilibrium and kinetics were simulated, too.

**Results:**

The optimum pH was found to be 6.0 at experimental conditions of initial copper(II) concentration 100 mg/L, biomass dose 2 g/L, contact time 12 h, shaking rate 150 r/min and temperature 25°C. Biosorption equilibrium cost about 1 hour at experimental conditions of pH 6.0, initial copper(II) concentration 100 mg/L, *C. lacerata* dose 2 g/L, shaking rate 150 r/min and temperature 25°C. At optimum pH 6.0, highest copper(II) biosorption amounts were 6.79 and 7.76 mg/g for initial copper(II) concentration of 100 and 200 mg/L, respectively (with other experimental parameters of *C. lacerata* dose 2 g/L, shaking rate 150 r/min and temperature 25°C). The pseudo second-order adsorptive model gave the best adjustment for copper(II) biosorption kinetics. The equilibrium data fitted very well to both Langmuir and Freundlich adsorptive isotherm models.

**Conclusions:**

Without further acid or alkali treatment for improving adsorption properties, formaldehyde inactivated *C. lacerata* biomass possesses good biosorption characteristics on copper(II) removal from aqueous solutions.

## Background

Heavy metal pollution is an increasing environmental problem of worldwide concern. Reducing heavy metals to environmentally acceptable limits in a cost-effective, easily available and environmental friendly manner becomes more and more urgent [1,2]. Biosorption of heavy metals from wastewaters by pretreated fungal biomass has gained growing acceptance since the 1990s [3,4].

The heavy metal ion biosorption by fungal biomass is based mainly on two mechanisms: covalent bonding with functional groups including carboxyl, hydroxyl, phosphate, amino, sulphydryl, and the result of physicochemical inorganic interactions directed by adsorption phenomena [2,5-7]. Therefore, here are several critical parameters affecting biosorption characteristics, such as pH, pretreatment methods, metal species, initial concentration of solutions, quantity of biomass, contact time [8-10].

Many fungi have been extensively studied and proved to be good biosorbents of heavy metals, such as *Rhizopus arrhizus* [11-14], *Aspergillus* spp. [6,15-17], *Penicillium* spp. [17,18] and *Saccharomyces* spp. [2,19]. However, white-rot fungi were relatively less reported for their biosorption though they were strong degrader of various xenobiotics and detoxicating materials of contaminated effluents [20,21]. They also possess the capacity of heavy metal biosorption [21].

*Ceriporia lacerata* is a white-rot fungus first isolated as a new species from white-rotted wood in Japan [22]. Till 2006, only four other reports published about it, referring to its taxonomy, genetics or decomposition [23-25]. Since 2007, *C. lacerata* has been more widely researched on its clinical significance, wood-decaying effect, metal tolerance and sorption potential and some other characteristics [26-30]. Kim et al. [31] found that the cadmium(II) removal rates by *C. lacerata* in stationary and shaking cultures were about 7% and 11%, respectively. However, there is so limited information yet available on this species that its other properties need further study. The objectives of this work were to verify the capacity of dead *C. lacerata* in copper(II) removal under batch conditions, to determine the influences of parameters involved, and to simulate the adsorptive equilibrium and kinetics.

## Materials and methods

### Preparation of the biomass

Fungus *C. lacerata* was isolated from the litter of *Solidago canadensis* (an exotic plant to China) in Pukou, Nanjing, China. It was cultivated at 25°C in 250 mL flasks containing 100 mL liquid medium composed of malt extract (20 g/L), peptone (1 g/L) and dextrose (20 g/L). After about 10 days incubation on a shaker at 150 r/min, *C. lacerata* mycelium was washed several times with deionized water, and then inactivated by immersion into 1% formaldehyde. After washing, the mycelium was dried at 60°C for 24 hour (h). Finally, dry mycelium was ground and sieved (mesh size < 0.5 mm).

### Metal solutions

Copper(II) solutions of 5 to 300 mg/L were obtained by diluting copper(II) stock solution (1 g/L), which was prepared by dissolving CuCl_2_ · 2H_2_O (analytical reagent grade, Shanghai Zhenxing Chemical Reagent Factory, China) in deionized water. Solution pH was adjusted with 0.1 mol/L HCl and NaOH and measured by pH meter (PHS-3C, Shanghai Hongyi Instrumentation Co., Ltd, China).

### Batch biosorption experiments

Batch biosorption experiments were conducted separately to evaluate the effects of pH, time, initial copper(II) concentration on biosorption of copper ions. All experiments were performed in duplicate, and the mean values were taken as the final results. For every treatment, 0.2 g dead biomass was added into 100 mL of copper(II) solution in 250 mL flask. The flasks were shaken (150 r/min) at 25°C for 12 h. Then, copper(II) solutions were vacuum filtered through Millipore membrane filters (0.45 μm, Shanhai Xingya Purification Material Factory, China). After dilution, initial and equilibrium copper(II) concentrations were determined using an atomic absorption spectrometer (AA320CRT, Shanghai Analytical Instrument Overall Factory, China). The copper(II) biosorption amount was calculated by Eq. (1):

1 $$ {q}_e=\frac{V\left({C}_0\hbox{-} {C}_e\right)}{m} $$

where *q*_*e*_ (mg/g) is the amount of copper(II) adsorbed on per gram of biosorbent, *V* (L) is the volume of copper(II) solution in the flasks, *C*_*0*_ and *C*_*e*_ (mg/L) are the initial and equilibrium copper(II) concentration, respectively, and *m* (g) is the dry weight of dead *C. lacerata* biomass.

Experiments to evaluate the effect of pH on biosorption were conducted constantly at pH 2.5 to 7.0, with intervals of 0.5, while initial copper(II) concentration was 100 mg/L.

Experiments to analyze the effect of contact time were operated at optimum pH and copper(II) concentration of 100 mg/L. Samples were harvested at 1/12, 1/6, 1/4, 1/2, 1, 2, 4, 6, 8 and 12 h.

Experiments to analyze the effect of initial sorbate concentration were performed at 5, 10, 25, 50, 75, 100, 200 and 300 mg/L (at optimum pH).

### Biosorption kinetics analysis

Kinetic models can simulate the data of contact time experiments. This study was simulated by both the linear first-order Lagergren (Eq. (2)) and pseudo second-order (Eq. (3)) models [6,32-34]:

2 $$ \ln \left({q}_e-{q}_t\right)= \ln {q}_e-k\hbox{'}t $$

3 $$ \frac{t}{q_t}=\left(\frac{1}{q_e}\right)t+\frac{1}{{2{k}_P{q}_e}^2} $$

where *k’* (h^−1^) and *k*_*P*_ (g/mg · h) are the first and second-order rate constants, respectively, *q*_*e*_ and *q*_*t*_ are the amounts of copper entrapped on per gram of biosorbent (mg/g) at equilibrium and time *t* (h), respectively.

### Biosorption isotherm analysis

Experiments to evaluate the effect of initial sorbate concentration were also used for biosorption isotherm studies. Langmuir and Freundlich isotherms were used to simulate the experimental data from the batch system at 25°C.

The linear Langmuir isotherm equation is:

4 $$ \frac{1}{q_e}=\frac{1}{q_{max}}+\left(\frac{1}{q_{max}{K}_L}\right)\frac{1}{C_e} $$

where *q*_*max*_ (maximum possible amount of copper adsorbed on per gram of biosorbent, mg/g) is the monolayer biosorption capacity of the biomass, and *K*_*L*_ is the Langmuir adsorption constant (L/mg) [21,35]. The equation of the Freundlich model is:

5 $$ {q}_e={K}_F{C_e}^n $$

where *K*_*F*_ and *n* are Freundlich adsorption constants and they respectively indicate adsorption capacity and intensity [10,36].

## Results and discussion

### Effect of pH on biosorption

**Figure 1:**
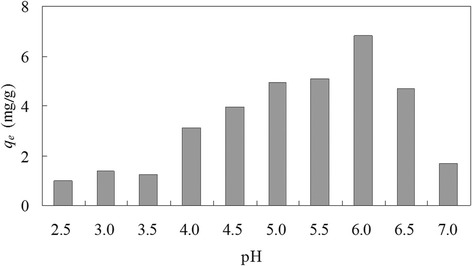
The effect of solution pH on copper biosorption by *Ceriporia lacerata*. Experimental conditions: initial copper(II) concentration = 100 mg/L, volume of copper(II) solution = 100 mL, *Ceriporia lacerata* dose = 2 g/L, contact time = 12 h, shaking rate = 150 r/min, temperature = 25°C.

Copper(II) biosorption capacity of dead *C. lacerata* biomass at different pH is shown in Figure 1. Approximately, the biosorption capacity of biomass increased with an increase of pH from 2.5 to 6.0, and then decreased at pH 6.5 and 7.0. Additionally, during the adsorptive process, the pH of equilibrium solution was slightly lower than that of initial solution.

Previous studies showed that pH value of the solution was an important parameter for both solution chemical properties of metals and surface characteristics of biosorbents [37-41]. According to Asmal et al. [42], there are three species of copper present in solution: Cu^2+^, CuOH^+^ and Cu(OH)_2_. At low pH (here maybe from 2.5 to 3.5), H^+^ ions competed with Cu^2+^ ions for the biosorption sites, that is protonation of the cell wall components negatively affected the biosorption capacity of dead *C. lacerata* biomass. However, this effect became less with the increase in pH (from 4.0 to 6.0) owing to that the raise of negative charges density on the cell surface offered more metal binding sites [6,7,15]. At this pH Cu^2+^ and CuOH^+^ were more favourable copper species. Therefore, just like the results of some works [6,43-45], we also found a sharp increase in biosorption with a slight increase of pH (at around pH 3.5). At higher pH (≥6.5), precipitation of copper(II) hydroxide occurred and precipitated on surfaces of biomass and bottle wall. Furthermore, all those above suggested that ion-exchange played an important role in biosorption of copper(II) ions by dead *C. lacerata*.

The optimum pH was 6.0 at which copper(II) biosorption capacity of dead *C. lacerata* biomass reached 6.79 mg/g. The optimum pH values of different reports on copper(II) biosorption by different biomasses differ quite a bit. *Phanerochaete chrysosporium* fungal biomasses [7,40] and three species of dead fungal biomasses (*Cladosporium cladosporioides*, *Gliomastix murorum* and *Bjerkandera* sp.) [46] showed the same optimum pH 6.0 for copper(II) removal, while cone biomass of *Thuja orientalis* showed the optimum pH value to be 7.7 [47] and *Chlorella vulgaris* algal biomass to be 5.0. The components and structural characteristics of various biomasses are quite diverse, which may be the most important reason for optimum pH differences. There must be other reasons such as different experimental parameters and operating error.

### Effect of contact time on biosorption

**Figure 2:**
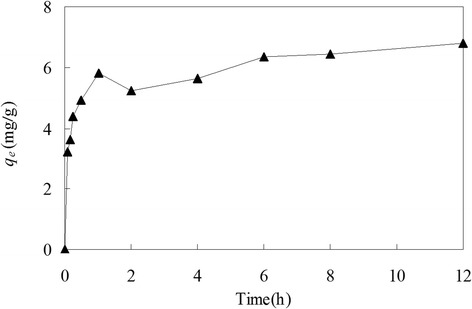
The amount of copper biosorption by *Ceriporia lacerata* at different contact time. Experimental conditions: pH 6.0, initial copper(II) concentration = 100 mg/L, volume of copper(II) solution = 100 mL, *Ceriporia lacerata* dose = 2 g/L, shaking rate = 150 r/min, temperature = 25°C.

At optimum pH, the amount of copper(II) adsorbed by dead *C. lacerata* increased during the biosorption process (Figure 2). This process consisted of two phases: the rapid phase during the first one hour at which biosorption contributed significantly great to adsorptive equilibrium, and the subsequent slower phase when biosorption contributed relatively small. At the end of the rapid phase, the amount of copper(II) biosorption reached 86% of the equilibrium which cost about 1 hour. The copper(II) biosorption decreased at the 2nd and 4th hours might because at that time the slightly decrease of pH resulted in H^+^ ions competing slightly with Cu^2+^ and CuOH^+^ ions for the biosorption sites and/or because of experimental errors, which needs to be further studied.

The equilibrium time of the copper biosorption by fungal biomass is determined by many parameters such as agitation rate of the solution, pretreated methods of the fungal biomass, structural properties and quantity of the biosorbents, the existence of other metal ions and initial copper(II) concentration [21]. Therefore, one species of biosorbent may cost different time (ranging from a few minutes to several hours [7,14,48]) to reach equilibrium under different conditions. One hour as the equilibrium time of copper biosorption in this study was scarcely reported before.

### Effect of initial copper(II) concentration on biosorption

**Figure 3:**
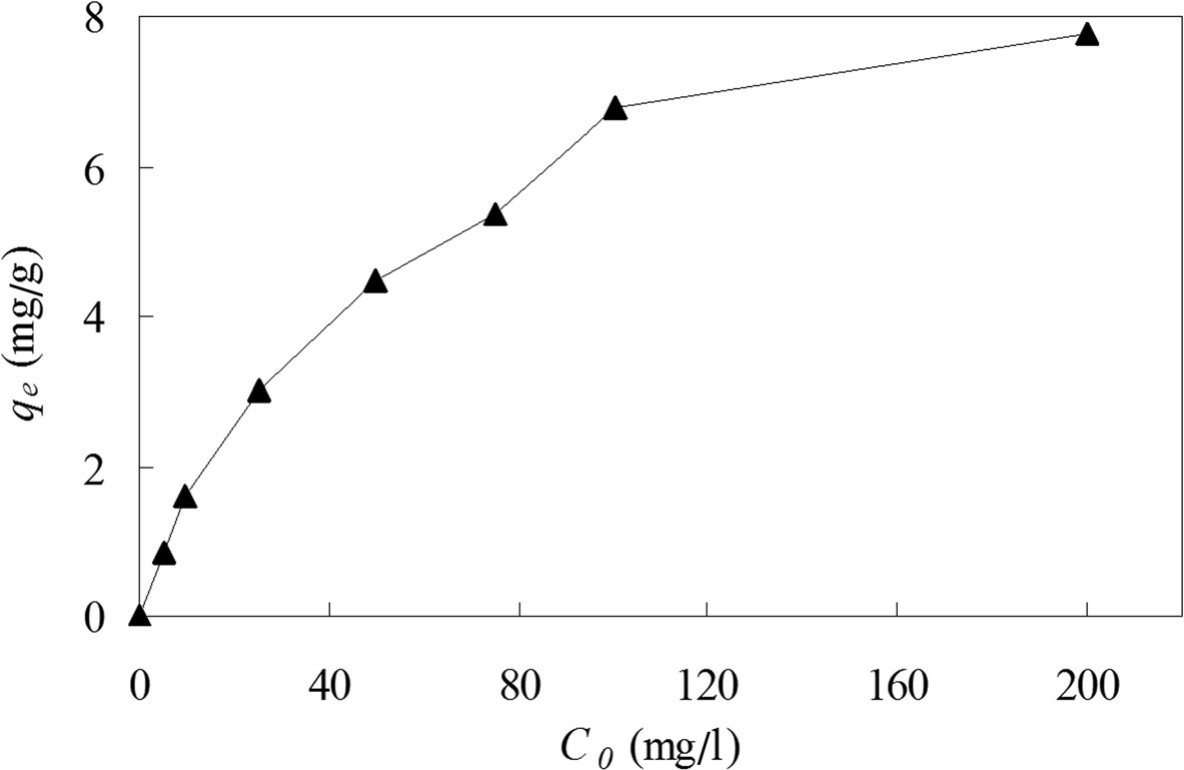
The effect of initial copper(II) concentration on copper biosorption by *Ceriporia lacerata* at optimum pH 6.0. Experimental conditions: pH 6.0, volume of copper(II) solution = 100 mL, *Ceriporia lacerata* dose = 2 g/L, contact time = 12 h, shaking rate = 150 r/min, temperature = 25°C.

The effect of initial copper(II) concentration on biosorption is presented in Figure 3. Approximately, copper biosorption increased with the increase of initial copper(II) ion concentration at the same unprecipitable pH. At pH 6.0, copper biosorption capacity increased sharply from 0.85 mg/g to 6.79 mg/g (Figure 3) while the initial copper(II) concentration was from 5 to 100 mg/L, but this increase became minor if the initial concentration continued to be raised. The highest biosorption capacity was 7.76 mg/g for 200 mg/L at pH 6.0.

At pH 6.0, copper(II) precipitated at initial concentrations higher than 300 mg/L. Under the same conditions, in the solutions of higher concentration, there were much more copper(II) ions around the active sites of *C. lacerata* biomass. Thus the adsorptive process could proceed more sufficiently, and that is why copper(II) adsorptive capacity increased with the increasing of initial concentration.

### Biosorption kinetics

**Figure 4:**
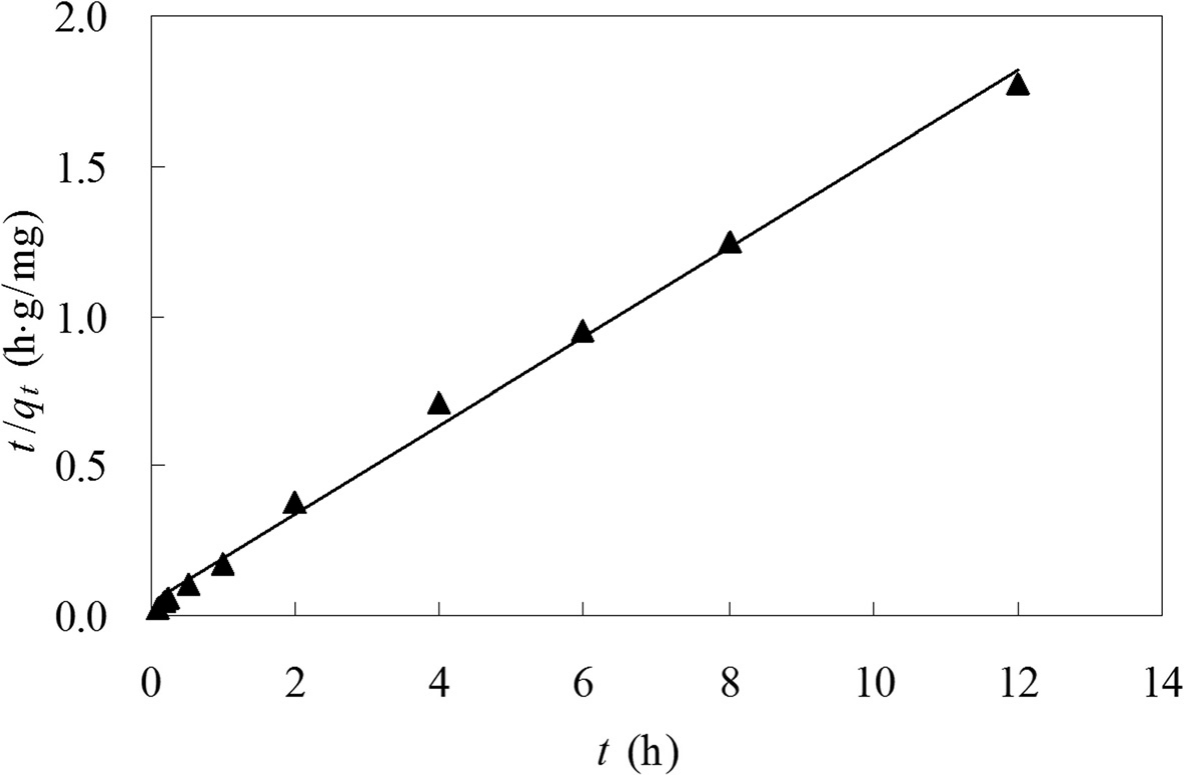
Linear plot of the pseudo second-order equation for copper biosorption by *Ceriporia lacerata*. Experimental conditions: pH 6.0, initial copper(II) concentration = 100 mg/L, volume of copper(II) solution = 100 mL, *Ceriporia lacerata* dose = 2 g/L, contact time = 12 h, shaking rate = 150 r/min, temperature = 25°C.

Mathematical models could be used for the quantitative description of the kinetic results. There were two kinetic equations very often used to simulate the metal ion adsorptive kinetics: the first-order Lagergren and the pseudo second-order rate equations [6,33;49]. In this study, the former with *q*_*e*_ (3.37 mg/g) and *R’*^2^ (0.8233) did not well fit experimental data, while the latter fitted the kinetic data with high regression coefficient (*R*_*P*_^2^, 0.9958) statistically significant (*p* < 0.05) (Figure 4 and Table 1). As a constant of pseudo second-order rate model, theoretical *q*_*e*_ (6.76 mg/g) was very close to the experimental *q*_*e*_ value (6.79 mg/g). That may be due to that biosorption is the rate-limiting step involving valence forces through sharing or exchanging electrons between biosorbent and sorbate [49]. In most cases (including this work), the former is applicable during the initial 1/3 or 1/2 hour but does not apply well throughout the whole process [49,50]. The pseudo second-order model was also proved most reliable by many researches [6,33,49,51].

**Table 1:** **Kinetic model constants for copper biosorption by** ***C*** **.** ***lacerata*** **at pH 6 and 25°C**

**Kinetic model**	**Constants of model**			
First-order Lagergren	*q*_*e*_ = 3.37 mg/g	*k’* = 11.05 × 10^−2^ h^−1^	*R’* ^2^ = 0.8233	*p* < 0.05
Pseudo second-order	*q*_*e*_ = 6.76 mg/g	*k*_*P*_ = 4.68 × 10^−4^ g/mg · h	*R*_*P*_^2^ = 0.9958	*p* < 0.05

### Biosorption isotherms

**Figure 5:**
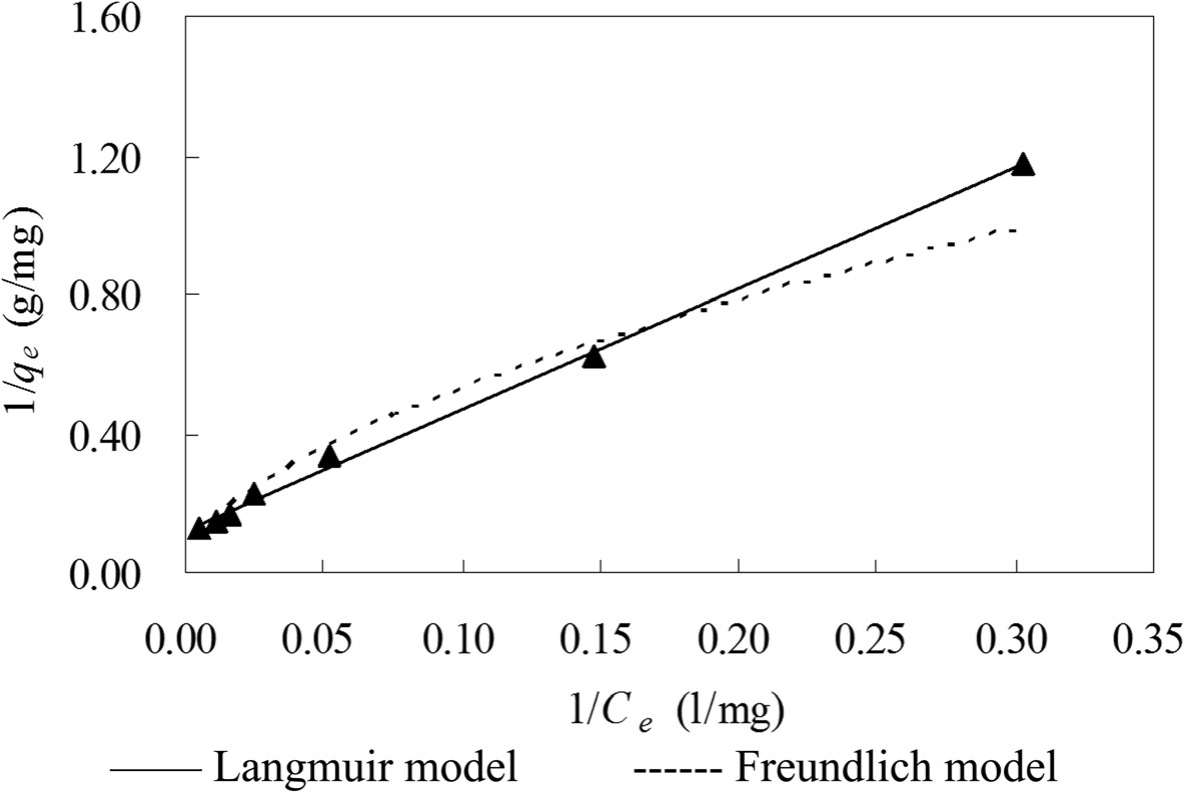
Copper biosorption isotherms by *Ceriporia lacerata* at pH 6 and 25°C. Experimental conditions: pH 6.0, initial copper(II) concentration = 5–200 mg/L, volume of copper(II) solution = 100 mL, *Ceriporia lacerata* dose = 2 g/L, contact time = 12 h, shaking rate = 150 r/min, temperature = 25°C.

Biosorption isotherm procedure assumes that all the external biosorption system parameters like pH and ionic strength are constant [52]. The Langmuir model assumes a monolayer adsorption of which energy is constant and no migration of sorbate molecules in the surface plane [2,5,35]. The Freundlich model is an empirical equation based on adsorption on a heterogeneous surface [36]. The former presented the better adjustment for copper(II) adsorption by dead *C. lacerata* than the latter did (Figure 5). The Langmuir model presented good adjustment with a regression coefficient (*R*_*L*_^2^) of 0.9979 (*p* < 0.05), and Freundlich model also gave a not bad adjustment with a *R*_*F*_^2^ value of 0.9696 (*p* < 0.05) (Figure 5 and Table 2).

**Table 2:** **Isotherm model constants for copper biosorption by** ***C*** **.** ***lacerata*** **at pH 6 and 25°C**

**Isotherm models**	**Constants of models**			
Langmuir	*q*_*max*_ = 8.31 mg/g	*K*_*L*_ = 3.45 × 10^−2^ mg^−1^	*R*_*L*_^2^ = 0.9979	*p* < 0.05
Freundlich	*n* = 0.56	*K*_*F*_ = 0.52	*R*_*F*_^2^ = 0.9696	*p* < 0.05

The Langmuir model showed that the maximum capacity of adsorbing copper(II) was 8.31 mg/g, which was assumed that at pH 6.0, 8.31 mg copper(II) would form a complete monolayer onto the surface of per gram of dead *C. lacerata*. The Freundlich model had constants of 0.52 for *K*_*F*_ value related to the adsorption capacity and 0.56 for *n* value related to the adsorption intensity.

### Comparison of biosorption capacity with other adsorbents

The maximum biosorption amount (*q*_*max*_) depends on fungal species, pretreating methods, the performed parameters. 8.31 mg/g as the ideal maximum value (*q*_*max*_) of copper(II) biosorption by dead *C. lacerata* was resulted at these conditions: initial copper(II) concentration of 100 mg/L, biomass 2 g/L, solution 100 mL, rotation 150 r/min and pH 6.0. The comparison of copper(II) adsorption capacities between dead *C. lacerata* biomass and other fungal biosorbents is shown in Table 3.

**Table 3:** **Copper(II) adsorption capacities (calculated from Langmuir constant** ***q***_***max***_ **) and experimental parameters of various fungal biosorbents from the literatures**

**Adsorbent**	**pH**	***C***_***e***_ **(mg/L)**	***q***_***max***_ **(mg/g)**	**References**
NaOH-treated *Aspergillus niger*	6.0	0-10	6.35	[6]
NaOH-treated *Botrytis cinerea*	5.0	5-300	20.35	[53]
Hydrochloric acid-treated waste beer yeast	5.0	3.2-44.8	1.46	[54]
Immobilized *Phanerochaete chrysosporium*	6.0	10-500	99.85	[40]
Dead *Pleurotus pulmonarius* (HCHO inactivated)	4.0	5-200	6.20	[10]
Dead *Schizophyllum commune* (HCHO inactivated)	4.0	5-200	1.52	[10]
Dead *Ceriporia lacerata* (HCHO inactivated)	6.0	5-200	8.31	This study

Pretreatments (taking NaOH-boiling and immobilization as examples) could avail copper(II) ions more functional groups to bind. That may be why formaldehyde inactivated *C. lacerata* biomass had lower biosorption capacity than those pretreated fungal biosorbents. Compared with other unpretreated fungal adsorbents, however, biosorption capacity of *C. lacerata* was relatively high.

## Conclusions

The results illustrated that formaldehyde inactivated *Ceriporia lacerata* biomass (without acid or alkali treatment for improving adsorption properties) showed a relatively high capacity in removal of copper(II) from aqueous solutions. The optimum operating conditions was proved to be at pH 6.0, contact time of 1 hour, initial copper(II) concentration of 200 mg/L. The pseudo second-order adsorptive model gave the best adjustment for copper(II) biosorption kinetics, while the equilibrium data fitted very well to both Langmuir and Freundlich adsorptive isotherm models. Without further acid or alkali treatment for improving adsorption properties, formaldehyde inactivated *C. lacerata* biomass possesses good biosorption characteristics on copper(II) removal from aqueous solutions. Prospectively, immobilized or further pretreated *Ceriporia lacerata* biomass has potential to be used as an efficient adsorbent in treatment of heavy metal polluted waters.
